# Force-velocity profile based training to improve vertical jump performance a systematic review and meta analysis

**DOI:** 10.1038/s41598-025-00870-1

**Published:** 2025-07-02

**Authors:** Paul Solberg, Will G. Hopkins, Vidar Andersen, Kolbjørn Lindberg, Thomas Bjørnsen, Atle Saeterbakken, Gøran Paulsen

**Affiliations:** 1 Norwegian Olympic and Paralympic Committee and Confederation of Sports, Oslo, Norway; 2 Western Norway University of Applied Sciences, Sogndal, Norway; 3 University of Agder, Kristiansand, Norway; 4 University of Stavanger, Stavanger, Norway; 5 Norwegian School of Sport Sciences, Oslo, Norway

**Keywords:** Testing of athletic performance, Strength and power training, Countermovement jump, Squat jump, Musculoskeletal system, Skeletal muscle

## Abstract

This systematic review and meta-analysis evaluated the effects of training optimized to correct deficits in vertical force–velocity (FV) profiles compared to non-optimized training. Outcomes included changes in the FV profile, vertical jump height, and maximal power. Searches followed PRISMA guidelines and were conducted in PubMed, Web of Science, SPORTDiscus, and Scopus. Study quality was assessed using the PEDro scale. As of March 2025, ten studies were identified; four were eligible for meta-analysis. Individually optimized FV-based training partially corrected a force deficit, fully corrected a velocity deficit, and had little effect on an already optimum FV profile. Effects on maximal power were small to trivial and often unclear when compared with non-optimized training. There were small-moderate improvements in jump height with optimized training, but these gains were comparable to non-optimized training. Heterogeneity was small to moderate, and methodological shortcomings were noted in all studies, including those excluded from the meta-analysis. Overall, it remains unclear if FV-profile-based training outperforms standard approaches. Labeling training “optimized” or “non-optimized” may induce placebo or nocebo effects, underscoring the need for blinded, randomized controlled trials.

## Introduction

Force–velocity (FV) profiles derived from multi-joint movements, such as vertical jumping, leg press, and sprinting, can be used to characterize athletes as force-dominant or velocity-dominant, or reciprocally, velocity-deficit and force-deficit. The implication is to correct the force or velocity imbalance through optimized (individualized) training and, in turn, augment performance^1^.

The vertical FV profiling concept was developed as a “simple” field method that combines the fundamental physics of accelerating a mass (Newton’s laws and energy conservation) with the inverse FV relationship of muscles^2,3^. An FV profile is a linear slope determined by F0 and V0 in an FV plot, where F0 is the theoretical force at zero velocity, and V0 is the theoretical maximal velocity at zero force^4^. An FV profile can be converted to a parabolic power profile with an apex that equals the maximal power (Pmax). Squat jumps (SJ) and countermovement jumps (CMJ) can both be used for vertical FV profiling^5^. Jumps can be performed with body weight (unloaded) and typically 2–5 external loads, such as a barbell across the shoulders (e.g., loads ranging from 20 to 80 kg). Jump height, body weight plus external load, and the push-off distance are the inputs used to derive averaged force and velocity values for each jump, and, in turn, the basis for the F0, V0, and Pmax variables, and the FV profile^2,6^.

According to Samozino, et al.^6^, an athlete can improve vertical jump performance via two mechanisms: increasing the Pmax and correcting the FV imbalance, where an optimal profile defines the latter. The optimal profile is based on a theoretical FV profile that maximally utilizes the power capability of the athlete in an unloaded (only body weight) vertical jump. The difference between the measured and optimal profiles yields the FV imbalance and classifies the athlete as force-deficient or velocity-deficient. If the measured profile is close to the optimal profile (< ± 10%), the athlete is deemed well-balanced^7^. The goal of FV-profile-based training is to achieve a well-balanced profile through optimized training.

Ten previous intervention studies included testing of the hypothesis that optimized FV-profile-based training is better than non-optimized training in improving vertical jumping^7–16^. Interestingly, these studies have shown mixed results. Some studies provide strong evidence that supports the hypothesis.^7,8^, while others have found that training opposite to – or against – the FV-profile recommendations worked equally well as training towards the optimal FV profile for improving jump performance^17^. Further, an improved correction of the FV imbalance without any improvements in vertical jump performance has also been observed^11^.

Jumping is a fundamental motor skill crucial in many sports^18^, and SJ and CMJ are among the most prevalent tests for assessing the mechanical capability of the lower limbs^19^. FV profiling has been an increasingly popular method for improving jump capacity among athletes, but the evidence is equivocal across published studies. Hence, this study aimed to conduct a systematic review to summarize the current evidence for vertical FV-profile-based training and synthesize the evidence in a meta-analysis of intervention studies that tested “optimized” FV-profile-based training against non-optimized training, i.e., similar to training for individuals with a well-balanced FV profile, without regard to the individuals’ FV profiles.

## Methods

### Literature search

**Fig. 1:**
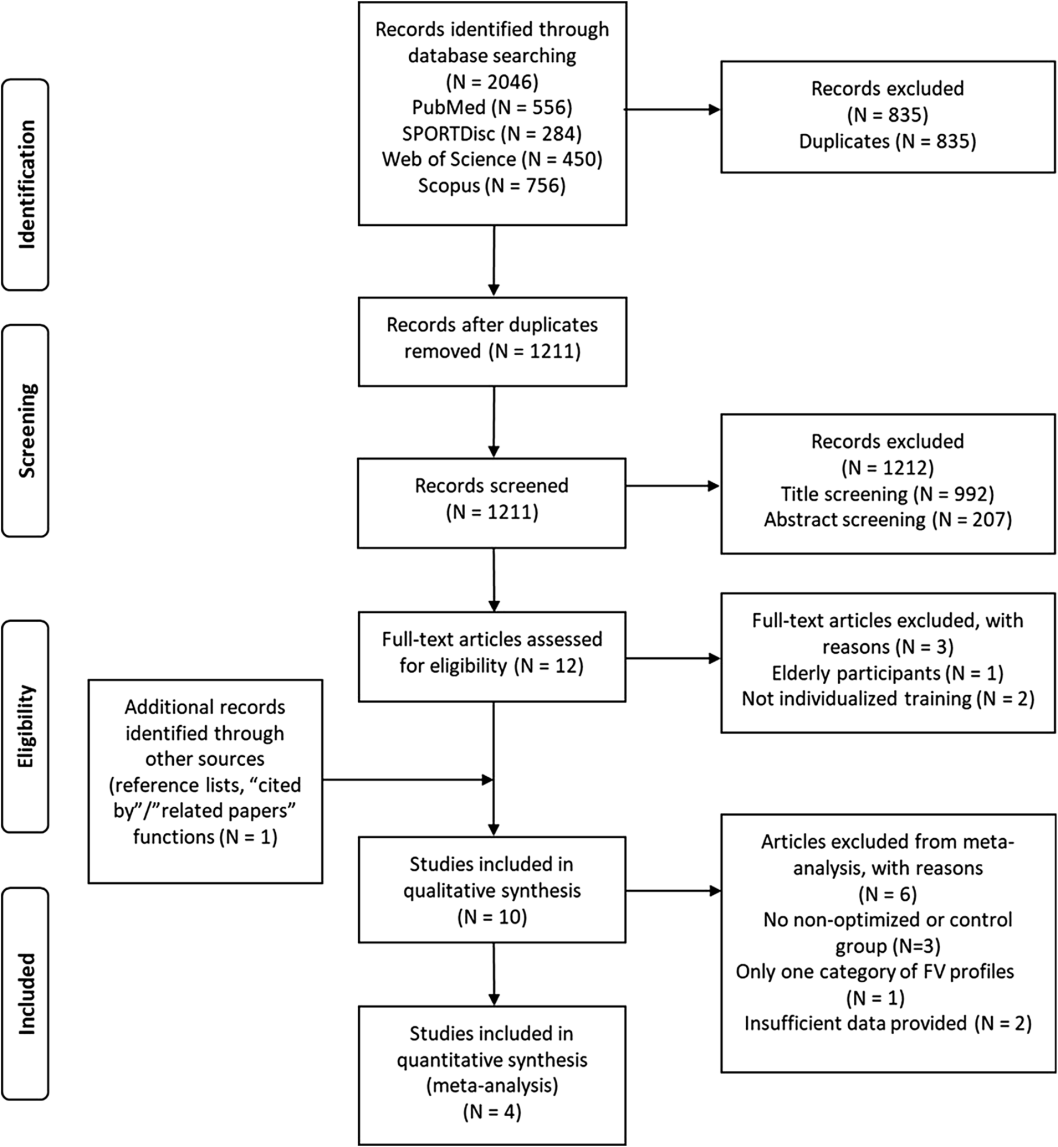
Preferred reporting items for systematic reviews and meta-analyses (PRISMA) flow diagram of the screening process.

A literature search was performed as outlined in Table 1, and the screening (PRISMA) procedure for the meta-analysis is provided in Fig. 1.

**Table 1:** Overview of the literature search procedure.

Category	Details
Databases searched	PubMed, Web of Science, SPORTDiscus, and Scopus
Search dates	Initial search May 2023, updated March 2025
Search conducted by	VA
Search terms (keywords and outcomes; Boolean search string)	(“force–velocity” OR “force velocity” OR “F-V-profile”) AND, (“individualized” OR “individualised” OR, “optimized” OR “optimised” OR, “training” OR “intervention” ) AND (“jumping” OR “jump height” OR vertical jump” OR “SJ” OR “CMJ” OR “squat*jump” OR counter*movement jump” OR “power output” OR Pmax” OR “maximal power” OR “FV-imbalance” OR “force–velocity imbalance ”)
Inclusion criteria	Intervention studies (> 4 weeks) utilizing vertical FV profiling, as described by Samozino, et al.^6^. Full-text peer-reviewed articles written in English
Exclusion criteria	Conference abstracts, theses, unpublished data, or non-English studies
Total studies identified	2046 studies
Duplicates removed	1211 studies removed; 835 studies screened after duplicate removal
Title and abstract screening by	VA and GP
Eligible for full-text analysis	10 studies eligible for full-text review
Additional screening methods	Reference lists examined; Google Scholar’s “Cited by” and “Related papers” functions used
Manual screening conducted by	VA, PS, and GP

### Selection criteria for the systematic review

In the systematic review, we wanted to include all intervention studies that employed vertical FV profiling with an optimal profile based on Samozino, et al.^6^. The criteria presented in Table 1 were based on the authors’ expertise and knowledge of the topic and the fact that only a few intervention studies existed.

### Selection criteria for the meta-analysis

In line with the PICOS framework^20,21^, our Population (P) comprised healthy athletes or strength-trained participants, as defined by the individual papers. The Intervention (I) involved using FV profiling to guide and assess training interventions intended to enhance vertical jump height. For the Comparison (C), studies included in the meta-analysis had to evaluate an “optimized” (i.e., individualized) FV-based training regimen against either a non-optimized (as for a well-balanced FV) protocol or a usual-practice control group; moreover, each study had to encompass at least two FV profile categories (force-deficit, well-balanced, or velocity-deficit). In terms of Outcomes (O), only those studies providing pre- and post-intervention vertical jump measures, together with sufficient data for analysis, i.e., standard error (SD), confidence intervals (CI), or exact p-values, were included. Finally, the Study Design (S) required randomized (controlled) trials or similarly rigorous designs suitable for meta-analysis, with clearly documented baseline and follow-up assessments linked to FV profiling (see Fig. 1).

Exclusion criteria encompassed the following: studies involving injured participants or those failing to meet the definition of healthy athletes or strength-trained individuals; studies that did not employ FV profiling to assess or guide training; studies lacking a non-optimized training group or control group; studies without baseline and follow-up vertical jump measures; studies lacking enough data to calculate effect sizes (e.g., missing jump-height outcomes or variance estimates); all publication types that did not present original data (abstracts, conference presentations, reviews, protocols, observational studies, and case reports).

Ten interventions were found eligible for review in the search process (see Fig. 1 and Table 2), but only four studies qualified for the meta-analyses^7,11,13,17^.

**Table 2:** The results from the PEDro scale assessment.

Authors	1*	2	3	4	5	6	7	8	9	10	11	Total score (0–10)	Rating
Included in the meta-analysis													
Jimenez-Reyes, et al.^7^	0	0	0	0	0	0	0	1	1	1	1	4	Fair
Zabaloy, et al.^11^	1	1	1	0	0	0	0	1	1	1	1	6	Good
Lindberg, et al.^17^	0	1	1	0	0	0	0	1	1	1	1	6	Good
Barrera-Dominguez, et al.^13^	0	1	1	1	0	0	0	1	1	1	1	7	Good
Not included in meta-analysis													
Álvarez, et al.^12^	0	0	0	0	0	0	0	1	1	1	1	4	Fair
Jimenez-Reyes, et al.^8^	0	0	0	0	0	0	0	1	1	0	1	3	Poor
Simpson, et al.^10^	0	0	0	0	0	0	0	1	1	1	1	4	Fair
Zubčić and Vučetić^14^	0	1	1	1	0	0	0	1	1	1	1	7	Good
Ishihara, et al.^16^	0	0	0	0	0	0	0	0	0	1	1	2	Poor
Conceição, et al.^15^	0	0	0	0	0	0	0	1	1	0	1	3	Poor

*Not included in the total score.

The six excluded studies were as follows:

1. Jimenez-Reyes, et al.^8^ included no non-optimized training or control groups. Moreover, the typical errors (TE) of jump height, which include individual responses, appeared unnaturally low compared to the TE in other studies.
2. Álvarez, et al.^12^ included only force-deficient participants. Moreover, the 95% CI reported was not centered around the mean change for CMJ jump height and was obviously wrong. An email was sent to the corresponding author, but no answer was given.
3. In Simpson, et al.^10^ the change scores were only available in figures, and the data could not be correctly extracted. We were unable to obtain the original data from the authors.
4. Zubčić and Vučetić^14^ had an inclusion criterion of including only force-deficient students (excluding or not finding well-balanced and velocity-deficient volunteers). The participants were university students, not athletes; no details about their training background were provided.
5. Ishihara, et al.^16^ included no non-optimized training or control groups. The reported vertical jump values were very high (> 70 cm), but this seems to be related to the measurement equipment used^22^.
6. Conceição, et al.^15^ included no non-optimized training or control groups. All the participants were force-deficient, and no group comparisons could be made.

### Quality of studies

Two researchers (PAS and VA) independently reviewed and rated the ten studies using the PEDro scale^23^. The PEDro scale includes 11 items assessing the methodological quality and bias risk in studies with a “yes” (1) and “no” (0). The studies were rated as poor quality (< 3), fair (4–5), good (6–8) and excellent (9–10)^24^. A total of seven items were rated differently between the two reviewers; these items were checked again, discussed, and agreed upon.

### Statistical analyses

A random-effects meta-analysis model was realized with the mixed-model procedure (Proc Mixed) in SAS Studio (Statistical Analysis System, version 9.4, Cary NC). For jump height and Pmax, the dependent variable was the log-transformed percentage change (100 × natural log[1 + percent change/100]). Sample estimates were weighted by the inverse square of their log-transformed standard errors, with the residual variance set to unity in the mixed model to perform the weighting^25^. Meta-analyzed mean effects were back-transformed to percent effects. Mean changes in FV balance and their standard errors were meta-analyzed without transformation. The standard errors were estimated from SD of change scores, or where they weren’t available, from exact *p* values or confidence limits. SD of change scores for the velocity-oriented group of Lindberg, et al.^17^ were imputed from the sample-size weighted mean of the SD of change scores in the other training groups in that study. The SD of change scores of FV balance in the well-optimized group of Zabaloy et al.^11^ was imputed as the sample size weighted mean of change scores of this measure in all the other studies. Owing to the limited number of study-estimates, a parsimonious mixed model was adopted, consisting of a nominal fixed effect for type of training (six levels: force-oriented, velocity-oriented, well-balanced, non-optimized, opposite-optimized, control) and random effects for study identity and sample-estimate identity within studies. The square root of the sum of study and sample-estimate variances provided an estimate of heterogeneity (the tau statistic, Higgins, et al.^21^), representing differences between settings in predicted mean effects not due to sampling variation. Standard errors of the variances were used to calculate appropriate lower and upper confidence limits for the heterogeneity, assuming a normal sampling distribution for the variances. None of the three measures showed evidence of potential outliers or publication bias in scatterplots of the random-effect solution versus standard errors of the study estimates^26^.

Magnitudes of effects were evaluated via standardization. For this purpose, a between-subject SD representing the typical variation of the given measure was derived from the square root of the weighted mean of the SD squared at baseline (where the weighting factor was the degrees of freedom of the SD in each study). The meta-analyzed effects were divided by this standard deviation, and their magnitudes were interpreted with the following scale: < 0.2, trivial; 0.2–0.6, small; 0.6–1.2, moderate; > 1.2, large^27^. The thresholds in this scale were halved for interpreting the magnitude of the standardized standard deviations derived from the random effects^28^. For jump height and Pmax, standardization was performed on the meta-analyzed log-transformed factor effects using log-transformed factor SDs, and the resulting standardized effects were adjusted for bias using a factor that is a function of sample size and magnitude of SD relative to the mean^29^. Standardized effects for FV balance were adjusted for small-sample bias with the usual factor^30^. Sampling uncertainty of the meta-analyzed effects is presented as ± 90% confidence limits and as quantitative chances of substantial and trivial magnitudes based on a Bayesian analysis with a minimally informative prior^31^. A probability (chances/100) and its complement (1 minus the probability) are *p* values for tests of the hypotheses that the effect has the given magnitude and does not have the given magnitude, respectively. An effect on mean jump height or maximal power was deemed to be unclear (inadequate precision) if it was potentially implementable (chance of benefit > 25%) but with an unacceptable risk of harm (> 0.5%); an effect of FV balance and the magnitude of the heterogeneity SD were deemed unclear if their confidence intervals included substantial positive and negative values. For effects with adequate precision, probabilities of substantial and/or trivial magnitudes > 25% were interpreted qualitatively with the following scale: 25–75%, possible, some or modest evidence for; 75–95%, likely, good evidence for; 95–99.5%, very likely, very good evidence for, clearly; > 99.5%, most likely, strong evidence for, clearly.

## Results

The systematic search is summarized in Supplementary Table 1.

### PEDro scale assessment and risk of bias

The PEDro scale assessment is presented in Table 2. The PEDro scores ranged from 2 to 7 with a median of 4.0. Four studies were interpreted as “good” methodological quality with a low risk of bias, three as “fair,” and three as “poor,” with a high risk of bias. None of the studies reported dropouts, applied intention-to-treat analyses, or reported whether the participants and researchers were blinded for treatment.

### Characteristic of the studies included in the meta-analysis

Four intervention studies were included in the meta-analysis, encompassing a total of 188 participants distributed across six different training intervention groups. Table 3 gives an overview of the groups and estimates.

**Table 3:** Total number of study estimates, total number of participants, and baseline values of the outcome measures in each group.

Group	Number of estimates	Number of participants	FV deficit^a^ Mean ± SD	JH (cm) Mean ± SD	Pmax (W kg^−1^) Mean ± SD
Force deficit	4	53	64 ± 20	31.1 ± 3.5	27.5 ± 5.9
Velocity deficit	3	25	132 ± 20	31.7 ± 4.3	23.7 ± 4.0
Well-balanced	3	18	99 ± 7	29.8 ± 3.5	22.8 ± 2.3
Non-optimized	3	37	84 ± 26	30.5 ± 4.0	23.3 ± 3.5
Opposite^b^	1	20	88 ± 21	32.7 ± 3.6	23.2 ± 3.8
Control^c^	2	35	74 ± 28	31.5 ± 4.4	24.6 ± 3.0

*FV* Force–velocity; *JH* Jump height; *Pmax* Maximal power; *SD* Standard deviation.

^a^100 represents a well-balanced profile, < 100 represents force deficit, > 100 represents velocity deficit.

^b^Participants trained away from, rather than towards, a balanced FV profile.

^c^Training as usual.

### Effects on the force–velocity profile

**Fig. 2:**
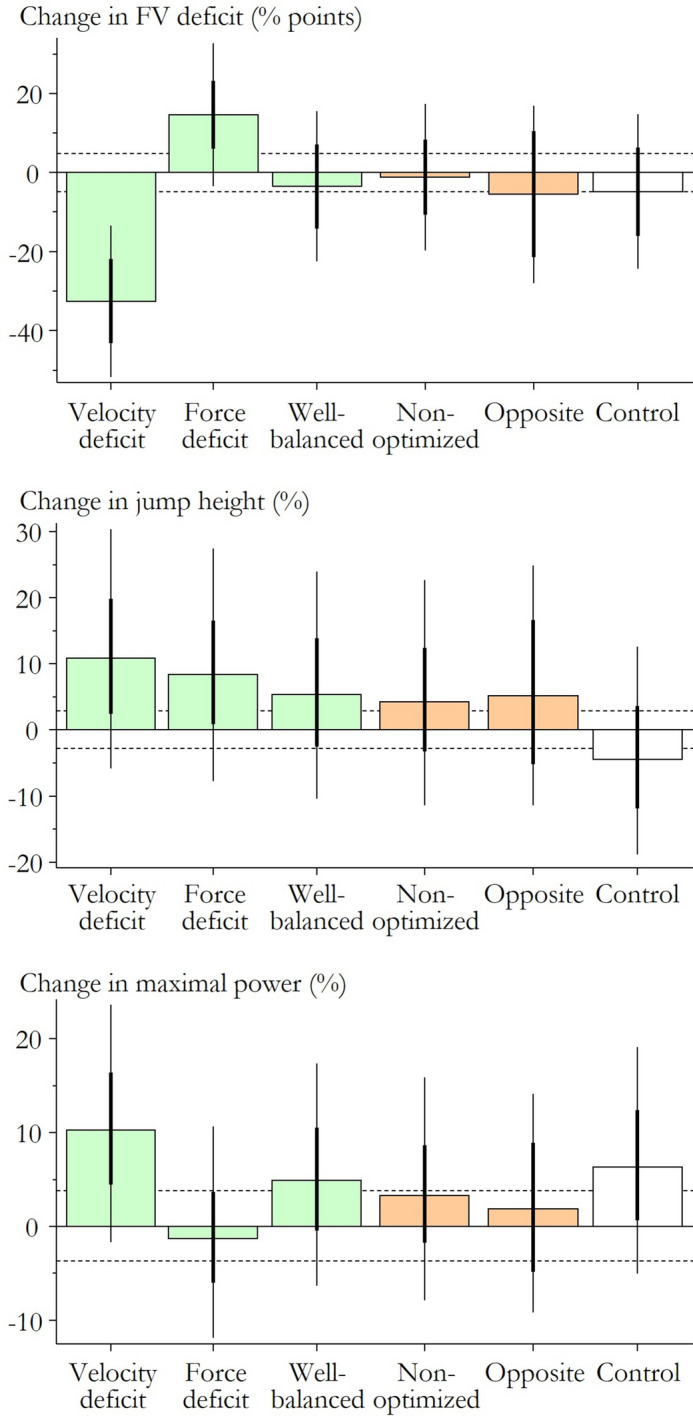
Meta-analyzed mean effects of the different types of training: green bars indicate optimized training based on the FV profile, orange bars indicate non-optimized and opposite-optimized training, and white bars are control (usual) training. The thick vertical lines are 90% confidence intervals for the overall mean effect across all possible settings (data also shown in Tables 3, 4, 5), while the thin lines indicate the 90% prediction interval (the range of values likely to contain the mean change in any new setting). The horizontal dotted lines are the smallest important positive and negative changes in the mean, demarcating trivial changes.

There were clear changes in the FV profile for those training to correct a velocity deficit (large, most likely negative) and a force deficit (moderate, very likely positive) (Fig. 2 and Table 4). Taking the baseline values into account, the velocity-deficit group was corrected completely (132 – 33 = 99% points, 100 representing well-balanced), while the force-deficit group was only partially corrected (64 + 15 = 79% points). Compared to controls, the effects of force and velocity deficit maintained their magnitude and precision. Training in the other groups produced little change in the FV profile, but these effects and comparisons with control were unclear.

**Table 4:** Predicted effects, comparisons with control, and heterogeneity in the meta-analysis of FV deficit.

	Effect (% points) Mean; 90% CI	Magnitude of observed effect^a^	Effect sign and probability^b^	Probability (%) ↓/ ↔ /↑
Predicted effects				
Velocity deficit	− 33; − 43 to − 22	Large	↓****	99.9/0.1/0.0
Force deficit	15; 6 to 23	Moderate	↑***	0.1/3.2/97
Well balanced	− 4; − 14 to 7	Trivial	↓ ↔	42/49/9.3
Non-optimized	− 1; − 11 to 8	Trivial	↓ ↔	25/62/14
Opposite	− 6; − 21 to 10	Small	↓ ↔	53/34/13
Control	− 5; − 16 to 6	Small	↓ ↔	50/43/7.2
Comparisons with control				
Velocity deficit	− 28; − 43 to − 13	Large	↓***	99/1.0/0.3
Force deficit	19; 6 to 33	Moderate	↑***	0.7/3.4/96
Well balanced	1; − 14 to 16	Trivial	↔ ↑	23/44/34
Non-optimized	4; − 11 to 18	Trivial	↔ ↑	15/42/44
Opposite	− 1; − 20 to 19	Trivial	↓ ↔ ↑	35/35/30
Heterogeneity SD (tau)	8; − 4 to 12	Moderate	↑	8/4/87

*CI* Confidence interval.

^a^Thresholds for small, moderate and large changes: ± 4.8%, ± 14% and ± 29%, respectively. Thresholds for heterogeneity are half these values.

^b^↑, ↔ , ↓ indicate effects that are at least possibly substantial positive, trivial, or negative, respectively. Probability of ↑ or ↓: *, possibly; **, likely; ***, very likely; ****, most likely. Probability of ↔ : ^0^, possibly; ^00^, likely; ^000^, very likely; ^0000^, most likely. These probabilities are shown for effects with adequate precision (chance of ↑ or ↓ < 5%).

Heterogeneity was moderate in observed magnitude and unclear (Table 4), but when combined with the mean changes, the resulting prediction intervals (Fig. 2) allow for clear substantial reduction in FV deficit with velocity-deficit training in any new setting, and possible substantial or trivial increases in FV deficit with force-deficit training in any new setting. The other types of training could result in substantial increases or decreases in FV deficit in new settings.

### Effects on jump height

For jump height, there were clear moderate and small likely beneficial changes in the velocity- and force-deficit groups, respectively (Table 5 and Fig. 2). Being in the control group had a small, possibly harmful effect on the jump performance.

**Table 5:** Predicted effects, comparisons with control, and heterogeneity in the meta-analysis of jump height.

	Effect (%) Mean; 90% CI	Magnitude of observed effect^a^	Effect sign and probability^b^	Probability (%) ↓/ ↔ /↑
Predicted effects				
Velocity deficit	10.8; 2.5 to 19.9	Moderate	↑**	0.7/5.2/94
Force deficit	8.4; 0.9 to 16.5	Small	↑**	1.4/8.8/90
Well balanced	5.4; − 2.5 to 13.9	Small	↑ ↔	4.5/25/71
Non-optimized	4.3; − 3.2 to 12.4	Small	↑ ↔	5.8/31/63
Opposite	5.2; − 5.1 to 16.6	Small	↑ ↔	9.6/26/65
Control	− 4.4; − 11.9 to 3.6	Small	↓*	65/29/6.2
Comparisons with control				
Velocity deficit	16.0; 6.0 to 26.9	Moderate	↑***	0.4/1.7/98
Force deficit	13.5; 4.9 to 22.8	Moderate	↑***	0.5/2.2/97
Well balanced	10.3; 0.9 to 20.6	Moderate	↑**	1.6/7.6/91
Non-optimized	9.1; 0.1 to 19.0	Moderate	↑**	2.1/9.5/89
Opposite	10.1; − 2.3 to 24.0	Moderate	↑**	4.4/12/84
Optimized^c^ vs. Others^d^	6.5; 1.1 to 12.1	Small	↑**	0.6/12/88
Optimized^c^ vs.Training^e^	3.3 − 3.0 to 10.0	Small	↑ ↔	5.4/41/54
Heterogeneity SD (tau)	6.9; − 3.4 to 10.6	Moderate	↑	8.8/2.1/89

*CI* Confidence interval.

^a^Thresholds for small, moderate, and large increases: 2.9%, 9.0%, and 19.0%, respectively. Corresponding thresholds for decreases: – 2.8%, – 8.3%, and – 16.0%. Thresholds for heterogeneity are approximately half these values.

^b^↑, ↔ , ↓ indicate effects that are at least possibly substantial positive, trivial, or negative, respectively. Probability of ↑ or ↓: *, possibly; **, likely; ***, very likely; ****, most likely. Probability of ↔ : ^0^, possibly; ^00^, likely; ^000^, very likely; ^0000^, most likely. These probabilities are shown for effects with adequate precision (benefit/harm odds ratio > 66 or chance benefit < 25%; chance of heterogeneity ↑ or ↓ < 5%).

^c^Mean of force deficit, velocity deficit, and well balanced.

^d^Mean of non-optimized, opposite, and control.

^e^Mean of non-optimized and opposite.

Because of the negative effect of the controls, the five different intervention groups all had a moderate, likely, or very likely beneficial, effect on jump height compared to the controls. The overall effect of optimal training was small and likely beneficial compared to non-optimal training and controls. When removing the controls, the effect of optimal training was small but unclear compared to non-optimal training.

Heterogeneity was unclear, but it represented moderate differences between study settings in the observed mean effects of each type of training (Table 5). The resulting prediction interval for each type of training spans substantial beneficial and harmful changes in jump height (Fig. 2), so the mean effects in a new setting are unclear.

### Effects on maximal power

There were clear small beneficial changes in maximal power following training in the velocity-deficit, well-balanced, and control groups, while there was a likely trivial effect for the force-deficit group (Fig. 2 and Table 6). The effects of non-optimized and opposite training were trivial and unclear.

**Table 6:** Predicted effects, comparisons with control, and heterogeneity in the meta-analysis of maximal power (Pmax).

	Effect (%) Mean; 90% CI	Magnitude of observed effect^a^	Effect sign and probability^b^	Probability (%) ↓/ ↔ /↑
Predicted effects				
Velocity deficit	10.2; 4.4 to 16.4	Small	↑***	0.2/3.6/96
Force deficit	− 1.3; − 6.0 to 3.7	Trivial	↔ ^00^	17/78/4.7
Well balanced	4.9; − 0.4 to 10.5	Small	↑*	1.0/35/64
Non-optimized	3.3; − 1.7 to 8.7	Trivial	↔ ↑	2.0/55/43
Opposite	1.8; − 4.8 to 8.9	Trivial	↔ ↑	8.0/62/30
Control	6.4; 0.7 to 12.4	Small	↑**	0.7/21/79
Comparisons with control				
Velocity deficit	3.6; − 2.8 to 10.5	Trivial	↔ ↑	3.6/49/48
Force deficit	− 7.2; − 11.9 to − 2.2	Small	↓**	90/10/0.4
Well balanced	− 1.4; − 7.1 to 4.7	Trivial	↔ ^0^	23/70/7.1
Non-optimized	− 2.8; − 8.3 to 3.0	Trivial	↔ ↓*	39/58/3.5
Opposite	− 4.3; − 11.5 to 3.5	Small	↓*	56/40/4.6
Optimized^c^ vs. Others^d^	0.7; − 3.0 to 4.5	Trivial	↔ ^00^	3.2/89/7.4
Optimized^c^ vs. Training^e^	1.9; − 2.6 to 6.6	Trivial	↔ ^00^	2.8/76/21
Heterogeneity SD (tau)	4.0; − 3.0 to 6.5	Small	↑	11/11/78

*CI* Confidence interval.

^a^Thresholds for small, moderate, and large increases: 2.9%, 9.0%, and 19.0%, respectively. Corresponding thresholds for decreases: – 2.8%, – 8.3%, and – 16.0%. Thresholds for heterogeneity are approximately half these values.

^b^↑, ↔ , ↓ indicate effects that are at least possibly substantial positive, trivial, or negative, respectively. Probability of ↑ or ↓: *, possibly; **, likely; ***, very likely; ****, most likely. Probability of ↔ : ^0^, possibly; ^00^, likely; ^000^, very likely; ^0000^, most likely. These probabilities are shown for effects with adequate precision (benefit/harm odds ratio > 66 or chance benefit < 25%; chance of heterogeneity ↑ or ↓ < 5%).

^c^Mean of force deficit, velocity deficit, and well balanced.

^d^Mean of non-optimized, opposite, and control.

^e^Mean of non-optimized and opposite.

Compared to controls, force-deficit and opposite training had a small likely and possibly harmful effect, respectively. The effect of the non-optimized training was trivial but possibly harmful, the effect of well-balanced training was possibly trivial, and velocity-deficit training induced a trivial and unclear effect. The effect of optimal training compared to non-optimal training was likely trivial both with and without the controls included.

The unclear heterogeneity represented small observed differences between study settings in the mean effects of each type of training (Table 6). The resulting prediction intervals (Fig. 2) show that velocity-deficit training could be beneficial but not harmful for maximal power in a new setting, while the other forms of training could be beneficial or harmful.

## Discussion

Herein, we investigated the effects of FV-profile-based training programs to improve the FV profile, vertical jump height, and maximal power. We found that optimized training was effective in correcting FV imbalances (deficits) and likely to be beneficial for improving jump height, with small (force-oriented) and moderate (velocity-oriented) effect sizes, but compared to control (training as usual), non-optimized, and opposite training, optimized force- and velocity-oriented training had similar, moderate benefits on jump height improvements. The six studies not included in the meta-analysis generally supported optimized training, albeit with methodological shortcomings.

Overall, the changes in vertical jump height after FV profile optimized training were ~ 8–11%, but for individual studies, the effects ranged from no effect^11^ to very large effects^7,13^. Jimenez-Reyes, et al.^7^ and Barrera-Dominguez, et al.^13^ reported that optimized training, on average, improved jump height by ~ 7–14% and ~ 21%, respectively. Outside the FV-profile literature, some strength/power and plyometric training studies have reported group-averaged changes in jump height up to ~ 20% in untrained individuals and ~ 15% in trained individuals, however, changes are most commonly reported in the range of 5–10%^32–36^. Consequently, the improvements in national-level basketball players (~ 7 cm; ~ 21%) observed by Barrera-Dominguez, et al.^13^, seem extraordinary.

The optimized training that displayed the largest effect size for jump height in the meta-analysis was velocity-oriented training (i.e., in velocity-deficient athletes). Velocity-oriented training was also effective in correcting the velocity imbalance and improving Pmax. Improved FV balance and Pmax have additive effects on jump height^6^. The efficacy of the velocity-oriented training may be a consequence of specific jump training^37^, as the velocity-oriented training programs typically contained a considerable volume of squat or countermovement jumps and combinations thereof^7^. The force-oriented training was partially effective in correcting the force deficit but improved jump height. However, force-oriented training led to a likely trivial change in Pmax, and the effect was likely harmful compared to control. These effects on Pmax are surprising since the training consisted of traditional heavy load strength exercises, such as the squat and deadlift (> 80% of 1 repetition maximum), combined with some power-oriented exercises, such as loaded jumps^7^. Heavy strength training combined with power training has been demonstrated to improve peak power and a rightwards shift of the FV relationship^38–42^. In Lindberg, et al.^17^, the improvements in jump height were modest, but across all training modalities, the improvement in jump height was associated with improved Pmax. In contrast, Barrera-Dominguez, et al.^13^ and Jimenez-Reyes, et al.^7^ observed an improved jump height but no changes in Pmax, which means that all of the improvement can be attributed to correcting the FV imbalance^6^.

The seemingly negative and harmful effect of optimized force-oriented training on Pmax could be due to an overestimation of Pmax in force-deficient athletes at baseline (pre-test) in some of the studies. The observation that force-oriented training apparently reduces V0 by as much as ~ 20%^7,12,13^ supports such a deduction. Since Pmax is calculated as F0*V0/4, a reduction in V0 equal to or larger than the increase in F0 will result in an unchanged or reduced Pmax. First, in force-deficient athletes, the Pmax is a theoretical value that falls outside the range of test loads as the load at Pmax allegedly is lower than body weight. Next, the observed magnitude of reductions in V0 is a physiologically very unlikely scenario because heavy strength training appears not to change or increase V0 on the muscle fiber level^43–45^. Theoretically, increases in the fascicle pennation angle and reduced fascicle lengths due to hypertrophy could hamper the maximal shortening velocity of the active muscles^46^. However, such negative effects appear unlikely, as they would require considerable hypertrophy, and strength training is generally more likely to increase fascicle length than reduce it^47^. Moreover, slow-velocity strength training does not appear to make muscles slower in single-joint movements^48,49^.

Giroux, et al.^49^ conducted a study where the plantar flexors were trained either with slow (high force) or fast (ballistic) velocity contractions. Both training protocols increased an estimated V0 for the plantar flexion. Interestingly, in an FV profile test based on SJ, the authors observed an increase in F0 concomitant with a substantial reduction in V0 after the slow training, highlighting the discrepancy of the isolated muscle properties and changes in the FV profile. Furthermore, possible neural adaptations to the heavy strength training are more likely to improve than reduce V0^50,51^. In a recent simulation study^52^, the authors explored how changes in the maximal force and shortening properties of the muscles affected F0 and V0 in the FV profile derived from SJ. The findings showed that by increasing either parameter, F0 and V0 increased concurrently, making an opposite effect physiologically implausible. From this, skill acquisition was the only logical explanation for an opposite change in F0 and V0. In other words, the athletes may be unfamiliar with jumping with heavy loads at the pre-test, which causes an underestimation of F0 and an overestimated V0. Heavy strength training and jump training with loads improve this specific motor skill, and the F0 is increased at the expense of V0. Such an outcome reflects the fact that F0 and V0 are mathematically interdependent when estimated via linear regression^6^– a relationship that does not mirror the physiological properties of skeletal muscle^53^. Hence, it highlights a fundamental shortcoming of the FV-profiling concept^52,54–56^. We acknowledge the V0 and F0 in the FV profile as purely theoretical values^3^, but it is practically misleading and confusing that V0 must be reduced at the expense of increases in F0 if Pmax is constant (or subject to relatively small changes).

In the control group, there was evidence for an increase in Pmax and a decrease in jump height. The finding was due mainly to the study by Barrera-Dominguez, et al.^13^, where the control group demonstrated an ~ 11% increase in Pmax. The change in the control group was not reported by Barrera-Dominguez, et al.^13^, but their Fig. 1 indicates a worsening of the FV imbalance over 8 weeks. Indeed, the worsening of the FV imbalance appears to be caused by an increased V0, which in turn causes an increased Pmax (as F0 was unchanged).

A potential factor that can affect the FV profiles is changes in body weight since FV variables are usually normalized to body weight. Notably, only Lindberg, et al.^17^ reported body weight measurements before and after the intervention, leaving it unclear whether body weight changes influenced the results in the other studies. Additionally, differing responses to individualized FV-profile-based training across studies may stem from other factors, such as variations in training programs, athlete skill levels, and the seasonal timing of interventions. The small to moderate observed heterogeneities in the present meta-analysis support this possibility (see further discussion below).

All the included studies have methodological shortcomings (Table 2 and Supplementary Table 1). Most importantly, none of the studies blinded the participants or the assessors to the intervention (at least, this is not stated in the papers). Placebo and nocebo effects may have contributed to the outcomes because the terms “individualized” and “optimized” on one side, contrasted with “non-optimized”, “opposite”, and “control” on the other, could create expectations of enhanced and impaired performance in the participants^57–59^. Interestingly, we observed that the control group experienced a harmful effect on jump height, which is consistent with a nocebo effect. In fact, we have recently demonstrated the importance of including a blinded placebo group in such studies^60^. For comparison, investigating the effects of altitude training may introduce similar issues, as altitude training is commonly regarded to be a form of optimized training^61^. Accordingly, it is indicated that athletes assigned to the “optimized” altitude training will experience a placebo effect, while the athletes assigned to the “non-optimized” sea-level control group will experience a nocebo effect^62^. Future studies should ensure that the results of the FV profile assessment and the assigned training are concealed for both the participants and the assessors. In addition, the participants’ expectations about the training should be assessed before and after the intervention to assess the efficacy of the blinding procedure^63^.

A second methodological issue is that only one of the ten studies identified in this systematic review and meta-analysis reported dropouts. Ishihara, et al.^16^ had 43% dropouts but gave no specific information about them. It is highly unlikely that there were no dropouts in the other studies, as 10–20% is a typical range in other domains^64,65^. Indeed, some athletes should, by sheer probability, have withdrawn due to the inherent risk of sports injuries^66^. Therefore intention-to-treat-analysis might have yielded different results.

We excluded six studies from the meta-analyses, owing to a lack of comparison groups, participant selections, errors in the results, unreasonably low standard errors, or lack of raw data. Consequently, with only four studies included, a major limitation was insufficient degrees of freedom to include baseline mean performance or other subject and study characteristics as effect modifiers. A further limitation was that only one heterogeneity could be estimated, representing the average heterogeneity across the different types of training. The resulting heterogeneities for the three outcomes were inconclusive, presumably because of differences in heterogeneity between the different types of training, along with uncertainty arising from the small number of study estimates, error of measurement of the outcome measures, the generally small sample sizes for some types of training, and/or the small number of study-estimates for some types of training. The observed values of heterogeneity were small to moderate, so with more studies, the heterogeneity SDs would likely be clearly substantial. Such heterogeneity would then, as alluded to above, be explained at least partly by effect modifiers representing differences between studies, for example, type of athlete and their performance level, training protocol (e.g., type of exercises and number of sessions), and test method^9,67^.

Of the studies identified in the systematic review but excluded from the meta-analysis^8,10,12,14–16^, all reported some positive effects of FV-profile-based training. Álvarez, et al.^12^ compared only against a training as-usual control group but observed large concurrent improvements in the FV imbalance and CMJ height (~ 15%), while the Pmax remained unchanged (which is a questionable finding, as discussed above). Álvarez et al. recruited ballet dansers and was the only study to investigate females. Notably, all the athletes were force-deficient and assigned primarily to heavy strength training. Jimenez-Reyes, et al.^8^ reported extremely consistent and effective improvements in the FV imbalance and SJ height but lacked both a non-optimized training group and a control group. Moreover, Jimenez-Reyes, et al.^8^ had a peculiar design where soccer and rugby players trained until they eventually reached a well-balanced FV profile (open-end training period). Simpson, et al.^10^ investigated rugby players and observed optimized training to improve SJ height and Pmax more than non-optimized training, but with small effect sizes. Zubčić and Vučetić^14^ recruited only force-deficient individuals (a force imbalance lower than 90% of the individual’s optimal profile). Their student participants (non-athletes) were allocated to a strength training group, i.e., optimized training, or a velocity-deficit group (“opposite training”, i.e., against the FV profile). While the velocity group had a non-significant 2% jump height improvement, the strength training group improved jump height by 12%. Intriguingly, Zubčić and Vučetić^14^ cited three papers when describing their velocity training protocol, which consisted of assisted jumping^68–70^. Unlike Zubčić and Vučetić^14^, all three papers reported that velocity training was effective, showing improvements in jump height of up to 10% in participants from a similar population. Hence, one could ask whether the lack of improvement in the “opposite” (velocity) group in the study by Zubčić and Vučetić^14^ reflects a possible nocebo effect. Ishihara, et al.^16^ followed American collegiate football players and observed that optimized training for force-deficient athletes induced a small improvement in jump height (4%), while optimized training for the velocity-deficient and well-balanced athletes caused no improvements. The Pmax was unaffected in all groups. Intriguingly, the heaviest athletes in the force-deficit group experienced a negative effect on their FV imbalance, i.e., they became more force-deficient during the intervention. It is also noteworthy that the athletes classified as “force deficient” in a 1 repetition maximum (1RM) test (thighs parallel to the ground) on average squatted 180 kg at the pre-test, which was similar to the well-balanced (169 kg) and the velocity-deficient (176 kg) groups. This highlights a seeming discrepancy between evaluating athletes with the FV procedure^6^ and 1RM tests, as these athletes, by most standards, will be considered very strong^71,72^. Yet, to be fair, a very strong athlete might theoretically jump higher if he/she becomes even stronger, but must then possess extraordinary velocity properties^6^. Finally, Conceição, et al.^15^ tested table-tennis players. After six weeks, the table tennis players were less force-deficient, and their jump height improved (~ 7%). The Pmax was unaltered, again as a consequence of increased F0 and decreased V0. In the study of Conceição, et al.^15^, all the players were force-deficient, and the authors included no sort of control or comparable training. All the athletes followed the same strength training-based program, and, hence, the study cannot really say anything about the concept of FV profile-based training. In short, it is striking that four of these six studies primarily demonstrate that heavy strength training improves jump height, as most of the participants were classified as force-deficient.

Only a few of the included studies investigated the training effects in more sport-specific tests, such as sprinting and agility tests. Some find no apparent transfer^10,11,16^, while others find a positive link^13,15^. This inconsistency aligns with generally poor correlations between the FV outcomes and other tests of neuromuscular properties and capabilities^9,11,73,74^.

Taken together, the present findings underscore that FV-profile-based training can yield improvements in jump height and correct FV imbalances, yet the magnitude and consistency of these effects vary widely among studies. As discussed, part of this variability may reflect a mix of skill acquisition, e.g., learning to jump effectively under specific load conditions, and actual changes in muscle properties. Reasonably, force-deficient athletes, who are exposed to heavy strength and power exercises (e.g., squats, deadlifts), are more likely to change their muscle properties^72^, while velocity-deficient athletes are more likely to correct the FV profile and improve jump height via specific skill improvements^52^. Of note, none of the included studies employed measurements of neuromuscular changes, such as hypertrophy, muscle architecture, or neural adaptations, leaving this unexplored and speculative.

## Conclusion

In the systematic review, we identified ten intervention studies. Eight of these studies claimed some degree of support for the effectiveness of FV-profile-based training but the absence of control groups and other methodological shortcomings provide overall weak evidence that FV-profile-based training is superior to non-optimized training. Only four studies qualified for the meta-analysis. We used meta-analyses to quantify the effects of FV-profile-based training on the FV profile, jump height, and maximal power of athletes. Training had its intended effects on the FV profile, but the resulting effects on maximal power in the various training groups were small to trivial and often unclear. The optimized training groups experienced small to moderate improvements in jump height similar to or a little better than those in non-optimized or opposite-optimized groups, but the uncertainties in the estimates preclude firm conclusions about any superiority of FV-optimized training. Given the observed negative effects of control training, we suspect substantial contributions of placebo and nocebo effects when interventions are framed as “optimized” (or “individualized”) vs. “non-optimized”. Future studies should employ a randomized controlled design where assessors and participants are blinded to the treatment conditions to properly assess the effects of training based on the FV profile. More studies are also needed to account for heterogeneity and its implications for the effectiveness of different training modalities in various settings. Additionally, underlying physiological mechanisms should be addressed. There is also a lack of studies on females, as 9 out of 10 studies included only males.

## Supplementary Information

Supplementary Information.

## Data Availability

The datasets used and/or analysed during the current study are available from the corresponding author upon reasonable request.

## Abbreviations

CI: Confidence interval
CMJ: Countermovement jump
F0: Force at zero velocity
FV: Force velocity
PEDro scale: Physiotherapy evidence database scale
Pmax: Maximal power
RM: Repetition maximum
SD: Standard deviation
SE: Standard error
SJ: Squat jump
TE: Typical error
V0: Velocity at zero force
